# Comparison of clinical characteristics between chronic bronchitis and non-chronic bronchitis in patients with chronic obstructive pulmonary disease

**DOI:** 10.1186/s12890-022-01854-x

**Published:** 2022-02-20

**Authors:** Joon Young Choi, Hyoung Kyu Yoon, Sang Yeub Lee, Jin Woo Kim, Hye Sook Choi, Yu-Il Kim, Ki-Suck Jung, Kwang Ha Yoo, Woo Jin Kim, Chin Kook Rhee

**Affiliations:** 1 Division of Pulmonary and Critical Care Medicine, Department of Internal Medicine, Incheon St. Mary’s Hospital, College of Medicine, The Catholic University of Korea, Seoul, Republic of Korea; 2 Division of Pulmonology, Critical Care and Sleep Medicine, Department of Internal Medicine, Yeouido St. Mary’s Hospital, College of Medicine, The Catholic University of Korea, Seoul, Republic of Korea; 3 Division of Pulmonology, Allergy and Critical Care Medicine, Department of Internal Medicine, Korea University Anam Hospital, Korea University College of Medicine, Seoul, Republic of Korea; 4 Division of Pulmonology and Critical Care Medicine, Department of Internal Medicine, Uijeongbu St. Mary’s Hospital, College of Medicine, The Catholic University of Korea, Uijeongbu, Republic of Korea; 5 Division of Pulmonary, Allergy and Critical Care Medicine, Department of Internal Medicine, Kyung Hee University Hospital, Seoul, Republic of Korea; 6 Division of Pulmonary Medicine, Department of Internal Medicine, Chonnam National University Hospital, Gwangju, Republic of Korea; 7 Division of Pulmonary, Allergy and Critical Care Medicine, Hallym University Sacred Heart Hospital, Hallym University Medical School, Anyang, Republic of Korea; 8 Division of Pulmonary, Allergy and Critical Care Medicine, Department of Internal Medicine, Konkuk University School of Medicine, Seoul, Republic of Korea; 9 Department of Internal Medicine and Environmental Health Center, School of Medicine, Kangwon National University, Chuncheon, Republic of Korea; 10 Division of Pulmonary and Critical Care Medicine, Department of Internal Medicine, Seoul St. Mary’s Hospital, College of Medicine, The Catholic University of Korea, 222 Banpodaero, Seochogu, Seoul, 06591 Republic of Korea

**Keywords:** Chronic obstructive pulmonary disease, Chronic bronchitis, Cohort study, KOCOSS database

## Abstract

**Background:**

Chronic bronchitis (CB) is associated with poor outcomes in patients with chronic obstructive pulmonary disease. The aim of this study was to identify the characteristics that distinguish chronic bronchitis (CB) from non-CB. In addition, the features of mild CB versus severe CB were compared and a cut-off level was defined according to CAT1 and CAT2 scores.

**Methods:**

This study was based on the Korea COPD Subgroup Study (KOCOSS) database, constructed in a multicenter COPD cohort study that recruited patients from 54 centers. CB was defined as CAT1 and CAT2 scores ≥ 3; severe CB was defined as CAT1 and CAT2 scores ≥ 4, while mild CB was defined as either a CAT1 or a CAT2 score < 4. Baseline characteristics, 1-year exacerbation rate, and 3-year FEV_1_ decline were compared in non-CB versus CB patients and in patients with mild CB versus severe CB.

**Results:**

Among the 2162 patients enrolled in this study, 497 (23%) had CB. These patients were more likely than non-CB patients to be current smokers; they also had higher symptom and depression/anxiety scores. Lung function tests showed lower FEV_1_, FEV_1_/FVC, and DLco values in CB patients. Among CB patients, 67.6% had mild disease. Symptom and depression/anxiety scores were worse in patients with severe CB than in patients with mild CB. There were no significant differences in the lung function tests of the two groups. Analysis of 1-year exacerbation rates in CB patients and non-CB patients revealed that patients with CB more frequently had moderate-to-severe exacerbations (OR = 1.46, *p* < 0.01). More severe exacerbation was also present in patients with severe CB than in patients with mild CB (OR = 2.52, *p* = 0.01). The difference in annual FEV_1_ decline rate did not significantly differ either between CB patients and non-CB patients or between patients with severe CB and patients with mild CB.

**Conclusions:**

CB patients had worse symptoms and lung function than non-CB patients; CB patients also had more frequent moderate-to-severe exacerbation. Patients with severe CB had higher symptom scores and more frequent severe exacerbation than did patients with mild CB.

**Supplementary Information:**

The online version contains supplementary material available at 10.1186/s12890-022-01854-x.

## Introduction

Chronic obstructive pulmonary disease (COPD) causes substantial morbidity and mortality worldwide, although it is a preventable and treatable disease [1]. Awareness of COPD heterogeneity is necessary for patient-tailored treatment [2, 3]. There have been wide accepted phenotypes including chronic bronchitis (CB), emphysema, asthma-COPD overlap (ACO), frequent/rare exacerbator and rapid decliner [4]. Of the known phenotypes, CB is the most well-understood. It is associated with typical symptoms, poor health-related quality of life, reduced lung function, frequent exacerbation, and high mortality [5, 6]. Furthermore, the economic burden of CB is substantial [7–9].

Patients with CB frequently have chronic cough and sputum [10], but the diverse disease definitions complicate outcome assessments [11–14]. In 1978, the American Thoracic Society defined CB based on the presence of frequent cough and sputum production for 3 months per year over 2 consecutive years; however, there were some limitations using in both clinical and research field [15, 16]. A recent approach to the assessment of CB is the use of sub-questionnaires from the COPD Assessment Test (CAT) [15, 17]. In the eight sub-questionnaires, the scores range from 0 to 5 points [18–20]. CAT1 and CAT2 evaluate the severities of cough and sputum, respectively; the combination of their scores is a valid approach to CB diagnosis. The CAT score also allows symptoms to be ranked based on a score of 0–5. By defining different CB cut-off levels, disease stratification according to severity may be possible, but this has not yet been attempted.

The aim of this study was to identify the characteristics that distinguish CB from non-CB within a group of COPD patients, based on baseline characteristics, symptoms, exercise capacity, lung function, and exacerbation rates. In addition, distinct CAT1 and CAT2 cut-off scores were used to stratify patients with mild CB versus severe CB. Forced expiratory volume in 1 s (FEV1) trajectories during a 3-year follow-up period were compared among non-CB, mild CB, and severe CB patients.

## Material and methods

### Study population and data collection

This study was based on the Korea COPD Subgroup Study (KOCOSS) database, constructed from a nationwide prospective cohort study—initiated in April 2012—that involved 54 medical centers in South Korea. The inclusion criteria were: age > 40 years and post-bronchodilator (FEV_1_)/forced vital capacity (FVC) ≤ 70% of the normal predicted value. The data were collected from case reports recorded by a doctor or trained nurse. After a baseline evaluation, patients were examined at 6-month intervals. For this study, data until November 2020 were extracted from the KOCOSS database and used to compare the clinical characteristics of non-CB, mild CB, and severe CB patients.

### Definition of CB and mild/severe CB

As recommended in previous studies, CAT1 (assessing cough) and CAT2 (assessing sputum) scores ≥ 3 were used to define CB [15, 21]. Patients with CAT1 and CAT2 scores ≥ 4 were considered to have severe CB; patients with either a CAT1 or a CAT2 score < 4 were considered to have mild CB.

### Clinical parameters

Baseline characteristics collected at the initial patient visit included age, sex, smoking history, and body mass index (BMI). Symptoms and functional exercise capacity scores were also obtained, including the modified Medical Research Council (mMRC) dyspnea score, the CAT score, and the 6-min walk distance test (6MWT) score. Additionally, the results of two psychological tests, the Beck Depression Inventory and the Beck Anxiety Inventory, were recorded.

Asthmatic features and markers associated with type 2 inflammation were analyzed, including history of asthma, asthma-COPD overlap (clinically diagnosed by a physician), fractional exhaled nitric oxide (FeNO), blood eosinophil count, and immunoglobulin E (IgE) level.

Pulmonary function test parameters (e.g., FEV1, FVC, FEV1/FVC, diffusion capacity of the lung for carbon monoxide [DLco], and residual volume/total lung capacity) were determined at baseline and annually for 3 years. Emphysema or bronchiectasis was diagnosed based on chest computed tomography (CT) findings interpreted by a radiologist. COPD medication regimens were categorized as long-acting beta-agonist or long-acting muscarinic antagonist, long-acting beta-agonist plus long-acting muscarinic antagonist, inhaled corticosteroid plus long-acting beta-agonist, and triple therapy. Both the occurrence and frequency of moderate-to-severe exacerbations and severe exacerbations during the first year of follow-up were analyzed. Moderate exacerbation was defined as a status requiring antibiotic or systemic corticosteroid therapy, administered in an outpatient clinic; severe exacerbation was defined as a status requiring an emergency room visit or hospital admission.

### Statistical analyses

All statistical analyses were performed using R software (ver. 3.6.3; R Development Core Team, Vienna, Austria). Continuous variables are expressed as means ± standard deviations; categorical variables are expressed as numbers and percentages. The above-listed clinical parameters were compared between non-CB patients and CB patients, and between patients with mild CB versus severe CB. Differences in the categorical and continuous values of two groups were determined using the χ^2^ test and Student’s *t*-test, respectively.

Negative binomial regression analysis was performed to predict the frequency of exacerbations in CB patients and non-CB patients. For the subgroup of CB patients, a regression model was used to predict the frequency of exacerbations in patients with severe versus mild CB. The regression models were adjusted for age, sex, smoking history, and post-bronchodilator FEV1. They were also used to analyze moderate-to-severe and severe exacerbations.

The annual FEV1 change over 3 years was assessed in a longitudinal analysis that used a linear mixed model in which the interaction was examined between time and CB. The interaction between time and the severity of CB (mild CB vs. severe) was also analyzed; the adjusted covariates were age, sex, and BMI.

## Results

### Differences in general characteristics between non-CB patients and CB patients

**Fig. 1:**
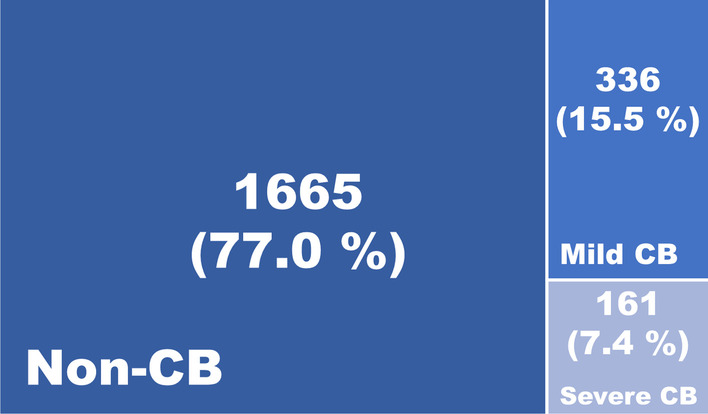
Distribution of non-CB, mild CB and severe CB

Of the 2162 COPD patients registered in the KOCOSS database between April 2012 and May 2021, 497 (23.0%) had CB as defined by the CAT score (both CAT1 and CAT2 ≥ 3) (Fig. 1). Differences in clinical characteristics between non-CB patients and CB patients are shown in Table 1. Compared with non-CB patients, CB patients were younger (69.2 ± 7.8 years vs. 68.3 ± 7.7 years, *p* = 0.02), more likely to be a current smoker (24.5% vs. 35.6%, *p* < 0.01), and had lower BMI (23.1 ± 3.4 vs. 22.6 ± 3.4, *p* < 0.01). There were no differences in the sex distribution. Symptom and functional exercise capacity scores were better in the non-CB group than in the CB group: mMRC (1.2 ± 0.8 vs. 1.6 ± 1.0, *p* < 0.01), total CAT score (12.0 ± 6.4 vs. 22.8 vs. 7.3, *p* < 0.01), and 6MWT (387.5 ± 117.5 m vs. 371.4 ± 109.8 m, *p* = 0.02). The two psychological scores showed that non-CB patients were less depressed (Beck Depression Inventory score, 6.1 ± 7.6 vs. 9.7 ± 9.7, *p* < 0.01) and less anxious (Beck Anxiety Inventory score, 3.8 ± 5.6 vs. 7.0 ± 9.1, *p* < 0.01) than CB patients. There were no significant differences in asthma history, asthma-COPD overlap, or type 2 inflammation markers (e.g., FeNO, blood eosinophil count, and IgE).

**Table 1:** Difference of clinical characteristics between non-CB and CB

	Non-CB (n = 1665, 77.0%)	CB (n = 497, 23.0%)	*p* value
Age	69.2 ± 7.8	68.3 ± 7.7	0.02
Sex (male)	1554 (93.3%)	462 (93.0%)	0.85
Smoking Hx			< 0.01
Never	119 (7.2%)	39 (7.8%)	
Ex-smoker	1135 (68.3%)	281 (56.5%)	
Current smoker	408 (24.5%)	177 (35.6%)	
BMI	23.1 ± 3.4	22.6 ± 3.4	< 0.01
mMRC	1.2 ± 0.8	1.6 ± 1.0	< 0.01
CAT score	12.0 ± 6.4	22.8 ± 7.3	< 0.01
6MWT	387.5 ± 117.5	371.4 ± 109.8	0.02
BDI score	6.1 ± 7.6	9.7 ± 9.7	< 0.01
BAI score	3.8 ± 5.6	7.0 ± 9.1	< 0.01
Asthma Hx	472 (28.6%)	158 (32.0%)	0.16
ACO	192 (21.1%)	71 (26.7%)	0.07
Emphysema	370 (44.0%)	133 (48.5%)	0.22
Bronchiectasis	97 (11.6%)	36 (13.1%)	0.55
Severity of airflow limitation (postBD FEV1%)			< 0.01
≥ 80%	181 (10.9%)	29 (5.4%)	
50–80%	877 (52.7%)	237 (47.7%)	
30–50%	492 (29.6%)	175 (35.2%)	
> 30%	114 (6.9%)	36 (11.3%)	
postBD FEV1 (L)	1.7 ± 0.6	1.6 ± 0.6	< 0.01
postBD FVC (L)	3.3 ± 0.8	3.3 ± 0.8	0.09
FEV1/FVC	50.8 ± 12.6	47.8 ± 12.7	< 0.01
DLco	64.8 ± 20.8	61.2 ± 20.3	< 0.01
RV/TLC	0.4 ± 0.1	0.4 ± 0.1	< 0.01
FeNO	26.8 ± 16.6	28.0 ± 18.3	0.70
Blood eosinophil count	225.2 ± 257.4	228.7 ± 216.9	0.79
IgE	239.5 ± 368.2	212.2 ± 296.4	0.27
Medications			
LABA or LAMA	440 (26.4%)	105 (21.1%)	0.02
LABA/LAMA	308 (18.5%)	76 (15.3%)	0.12
ICS/LABA	195 (11.7%)	58 (11.7%)	1.00
ICS/LABA/LAMA	365 (21.9%)	118 (23.7%)	0.43
M–S exacerbation (Y/N)	464 (37.5%)	177 (51.6%)	< 0.01
MS exacerbation (frequency)	0.9 ± 1.8	1.6 ± 2.6	< 0.01
S exacerbation (Y/N)	124 (10.0%)	45 (13.1%)	0.12
S exacerbation (frequency)	0.1 ± 0.5	0.3 ± 1.0	< 0.01

Data are presented as n (%) or mean ± SD

*BMI* body mass index, *BDI* Beck Depression Inventory, *BAI* Beck Anxiety Inventory, *mMRC* modified Medical Research Council, *CAT* COPD Assessment Test, *6MWT* 6-min walk distance test, *ACO* asthma-COPD overlap, *LAMA* long-acting muscarinic antagonist, *LABA* long-acting beta2-agonist, *ICS* inhaled corticosteroids

CT results were retrieved from total of 1171 patients in this study. The radiologic findings showed no significant differences between groups in presence of emphysema or bronchiectasis. Lung function tests showed that, compared with non-CB patients, CB patients had lower post-bronchodilator FEV1 (1.7 ± 0.6 vs. 1.6 ± 0.6, *p* < 0.01), lower FEV1/FVC (50.8 ± 12.6 vs. 47.8 ± 12.7, *p* < 0.01), and lower DLco (64.8 ± 20.8 vs. 61.2 ± 20.3, *p* < 0.01); thus, CB patients had more severe airflow limitations. Mono-bronchodilators were more often prescribed to non-CB patients (26.4% vs. 21.1%, *p* = 0.02); the prescription rates of dual bronchodilators, inhaled corticosteroid/long-acting beta-agonist, and triple agents did not significantly differ between groups. According to the baseline moderate-to-severe exacerbation history in the previous year, both the occurrence rate and the frequency were significantly higher in CB patients than in non-CB patients (51.6% vs. 37.5%, *p* < 0.01 and 1.6 ± 2.6 vs. 0.9 ± 1.8, *p* < 0.01); according to the baseline severe exacerbation history, there was no significant difference in the occurrence rate but CB patients had more frequent exacerbations (0.3 ± 1.0 vs. 0.1 ± 0.5, *p* < 0.01). The general characteristics of non-CB patients and CB patients among ever-smokers are shown in Additional file 1: Table S1. 

### Differences in general characteristics of mild CB versus severe CB

Among the 497 CB patients, 336 (67.6%) had mild disease and 161 (32.4%) had severe disease (Fig. 1). The clinical characteristics of these two groups are presented in Table 2. There were no significant differences in age, sex, smoking history, or BMI. The symptom and functional exercise capacity scores were consistent with an unfavorable outcome in the severe versus mild CB groups: mMRC (1.8 ± 1.1 vs. 1.5 ± 0.9, *p* < 0.01), total CAT score (27.3 ± 6.8 vs. 20.7 ± 6.5, *p* < 0.01), and 6MWT (353.0 ± 110.9 m vs. 379.9 ± 108.5 m, *p* = 0.03). The psychological scores indicated more depression and anxiety in the severe CB group than in the mild CB group (Beck Depression Inventory score: 11.4 ± 10.6 vs. 8.8 ± 9.1, *p* = 0.04; Beck Anxiety Inventory score: 10.5 ± 11.6 vs. 5.2 ± 7.0, *p* < 0.01). There were no significant differences between groups with respect to asthma history, asthma-COPD overlap, and markers of type 2 inflammation (e.g., FeNO, blood eosinophil count, and IgE level).

**Table 2:** Difference of clinical characteristics between mild CB and severe CB

	Mild CB (n = 336, 67.6%)	Severe CB (n = 161, 32.4%)	*p* value
Age	68.4 ± 7.8	67.9 ± 7.4	0.53
Sex (male)	313 (93.2%)	149 (92.5%)	0.95
Smoking Hx			0.41
Never	27 (8.0%)	12 (7.5%)	
Ex-smoker	196 (58.3%)	85 (52.8%)	
Current smoker	113 (33.6%)	64 (39.8%)	
BMI	22.7 ± 3.3	22.3 ± 3.5	0.30
mMRC	1.5 ± 0.9	1.8 ± 1.1	< 0.01
CAT score	20.7 ± 6.5	27.3 ± 6.8	< 0.01
6MWT	379.9 ± 108.5	353.0 ± 110.9	0.03
BDI score	8.8 ± 9.1	11.4 ± 10.6	0.04
BAI score	5.2 ± 7.0	10.5 ± 11.6	< 0.01
Asthma Hx	104 (31.2%)	54 (33.8%)	0.65
ACO	50 (29.1%)	21 (22.3%)	0.30
Emphysema	90 (48.9%)	43 (47.8%)	0.96
Bronchiectasis	23 (12.5%)	13 (14.4%)	0.80
Severity of airflow limitation (postBD FEV1%)			0.76
≥ 80%	18 (5.4%)	11 (6.8%)	
50–80%	165 (49.1%)	72 (44.7%)	
30–50%	117 (34.8%)	58 (36.0%)	
> 30%	36 (10.7%)	20 (12.4%)	
postBD FEV1 (L)	1.6 ± 0.6	1.5 ± 0.5	0.32
postBD FVC (L)	3.3 ± 0.8	3.2 ± 0.9	0.36
FEV1/FVC	47.8 ± 12.9	47.8 ± 12.4	0.99
DLco	61.7 ± 20.8	60.1 ± 19.2	0.46
RV/TLC	0.4 ± 0.1	0.4 ± 0.1	0.90
FeNO	30.5 ± 20.5	22.0 ± 9.8	0.08
Blood eosinophil count	228.2 ± 212.7	229.8 ± 225.8	0.95
IgE	223.3 ± 280.2	191.4 ± 325.4	0.46
Medications			
LABA or LAMA	77 (22.9%)	28 (17.4%)	0.20
LABA/LAMA	51 (15.2%)	25 (15.5%)	1.00
ICS/LABA	37 (11.0%)	21 (13.0%)	0.61
ICS/LABA/LAMA	79 (23.5%)	39 (24.2%)	0.95
M–S exacerbation (Y/N)	114 (47.9%)	63 (60.0%)	0.051
MS exacerbation (frequency)	1.4 ± 2.4	1.9 ± 2.9	0.13
S exacerbation (Y/N)	22 (9.2%)	23 (21.9%)	< 0.01
S exacerbation (frequency)	0.2 ± 0.9	0.4 ± 1.1	0.07

Data are presented as n (%) or mean ± SD

*BMI* body mass index, *BDI* Beck Depression Inventory, *BAI* Beck Anxiety Inventory, *mMRC* modified Medical Research Council, *CAT* COPD Assessment Test, *6MWT* 6-min walk distance test, *ACO* asthma-COPD overlap, *LAMA* long-acting muscarinic antagonist, *LABA* long-acting beta2-agonist, *ICS* inhaled corticosteroids

Patients with mild CB versus severe CB did not significantly differ in chest CT findings concerning emphysema or bronchiectasis, nor in terms of lung function test results and medication use. There were no significant differences in rate and frequency of moderate-to-severe exacerbations between severe CB and mild CB groups. Severe exacerbation occurred in a significantly larger number of patients with severe CB, compared with patients who had mild CB (21.9% vs. 9.2%, *p* < 0.01). However, there were no significant differences in the frequency of severe exacerbations between severe CB and mild CB group (0.4 ± 1.1 vs. 0.2 ± 0.9, *p* = 0.07). The general characteristics of patients with mild CB vs. severe CB who were ever-smokers are presented in Additional file 1: Table S2.

### Association of chronic bronchitis and frequency of exacerbation

Negative binomial regression analysis revealed an association between the frequency of exacerbation and CB (Table 3). Compared with non-CB patients, patients with CB had a larger annual number of moderate-to-severe exacerbations (OR = 1.46, 95% confidence interval, 1.20–1.79, *p* < 0.01). The frequency of severe exacerbation did not significantly differ between groups. Regression analysis to explore an association between CB severity and the frequency of exacerbation (Table 4) showed a significant relationship for severe exacerbation but not for moderate-to-severe exacerbation. The same results were obtained in the group of ever-smokers, although CB was also significantly associated with the frequency of severe exacerbation (OR = 1.52, p = 0.04) (Additional file 1: Tables S3, S4).

**Table 3:** Frequency of exacerbations for CB compared with non-CB patients

	Moderate-to-severe exacerbation			Severe exacerbation		
	OR	95% CI	*p* value	OR	95% CI	*p* value
CB	1.46	1.20–1.79	< 0.01	1.40	0.96–2.03	0.08
Age	1.00	0.99–1.01	0.87	1.00	0.97–1.02	0.81
Sex (male)	0.76	0.53–1.09	0.13	0.40	0.19–0.81	0.01
Smoking Hx	1.06	0.89–1.26	0.52	0.96	0.68–1.36	0.81
FEV1	0.36	0.31–0.43	< 0.01	0.16	0.11–0.22	< 0.01

*CB* chronic bronchitis

**Table 4:** Frequency of exacerbations for severe CB compared with mild CB

	Moderate-to-severe exacerbation			Severe exacerbation		
	OR	95% CI	*p* value	OR	95% CI	*p* value
Severe CB	1.29	0.92–1.82	0.14	2.52	1.23–5.32	0.01
Age	1.01	0.98–1.03	0.62	1.02	0.97–1.06	0.51
Sex (male)	0.70	0.34–1.45	0.33	0.19	0.03–1.11	0.08
Smoking Hx	1.13	0.83–1.55	0.42	0.91	0.44–1.92	0.80
FEV1	0.36	0.27–0.49	< 0.01	0.08	0.03–0.17	< 0.01

*CB* chronic bronchitis

### Association of chronic bronchitis and lung function change

**Fig. 2:**
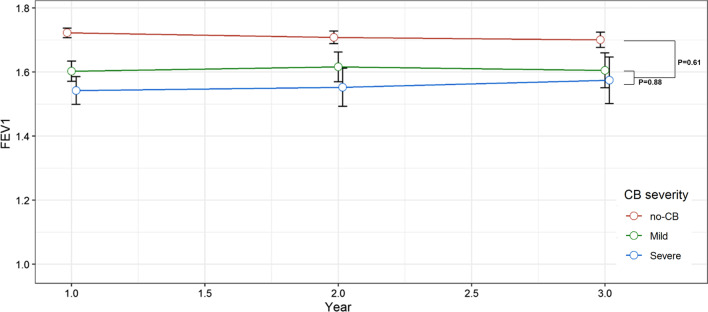
FEV1 trajectories of non-CB, mild and severe CB

Among 2162 patients included in this study, every patients received lung function test in the baseline visit, 1157 in the first, 769 in the second and 507 in the third year of the follow-up. Linear mixed model analysis revealed no significant differences in the annual rate of FEV1 change either between non-CB patients and CB patients (+ 2 ml/year vs. + 8 ml/year, *p* = 0.61) (Fig. 2) or between patients with mild CB versus severe CB (+ 2 ml/year vs. + 6 ml/year, *p* = 0.88).

## Discussion

Our analysis of a nationwide COPD cohort database revealed differences in clinical characteristics between non-CB patients and CB patients. By stratifying CB patients into patients with mild disease and patients with severe disease based on CAT sub-questionnaires, we were able to quantify the degrees of symptoms. The results showed that, compared with patients who had mild CB, patients with severe CB had worse outcomes in terms of respiratory symptoms, functional exercise capacity, and depression and anxiety scores; they also had more frequent severe exacerbations.

The clinical significance of the CB phenotype in COPD is well-established. CB is associated with poor health-related quality of life [22–25], poor functional exercise capacity [26], low lung function [11, 22, 23, 27, 28], more frequent exacerbations [11, 17, 22, 23, 27], and higher mortality [24, 29]. Consistent with those previous findings, our study showed higher mMRC and CAT scores, worse 6MWT performance, worse lung function, and more frequent exacerbations in CB patients than in non-CB patients. In most studies, including ours, CB patients were younger and had lower BMI; however, conflicting results have also been reported [11, 23, 26, 27, 30]. Furthermore, in our study and other studies, CB was strongly associated with a history of smoking [11, 23, 31]. In a study concerning the association of CB with mental health, Meek et al. found poor outcomes for CB patients in terms of emotional and mental health sections of the 36-Item Short Form Health Survey (SF-36). Their assessment of SF-36 items related to depression showed that CB patients had higher scores for all of those items. Our CB patients also had higher depression and anxiety scores, compared with non-CB patients.

To our knowledge, the present study is the first to stratify CB patients according to disease severity, then to analyze the clinical characteristics of patients with mild CB versus severe CB. A previous study classified the severity of cough and sputum by using CAT1 and CAT2 scores [32]. Similar to our findings, the previous study showed that patients with more severe cough or sputum had higher mMRC scores and more frequent exacerbations. Patients with severe symptoms also had poor outcomes in terms of anxiety, depression, fatigue, physical function, social ability, sleep disturbance, and pain interference, as determined using the Patient-Reported Outcome Measurement Information System Scores (PROMIS-29). These results are consistent with our findings and highlight the broader implications of severe cough and sputum in patients with severe CB. In our study, there were no significant differences in baseline characteristics (age, sex, and BMI) between patients with severe versus mild CB; while patients with severe CB were more likely to be current smokers, the difference between groups was not statistically significant. However, patients with severe CB experienced more frequent severe exacerbations than did patients with mild CB (OR = 2.52); thus, patients with severe CB should be more carefully monitored.

Although our study showed worse lung function in CB patients than in non-CB patients, the 3-year follow-up data showed no significant differences in the FEV1 decline rate between groups. Similar results were obtained in patients with mild CB versus severe CB. However, according to the Coronary Artery Risk Development in Young Development in Young Adults (CARDIA) study, a prospective cohort study that repeatedly measured lung function in young adults over 30 years, the presence of cough or sputum was significantly associated with an excess annual decline in both FEV1 (− 2.71 ml/year, *p* < 0.01) and FVC (− 1.94 ml/year, *p* = 0.03) [33]. In the Rotterdam Study, the rate of FEV1 decline during a median 6.5 years of follow-up was higher in CB patients (− 38 ml/year, *p* = 0.02) [23]. The Copenhagen City Heart Study evaluated 5-year lung function test results and found excessive FEV1 decline in men with chronic mucus hypersecretion (22.6 ml/year, 95% confidence interval, 8.2–37.4) [34]. Thus, our follow-up interval of 3 years may have been insufficient to detect statistically significant differences between CB patients and non-CB patients. Moreover, 3-year follow-up data regarding FEV_1_ were available for only 24% of our patients, which may have led to bias in the results. Further studies involving longer durations may demonstrate that disease severity is an important factor contributing to lung function decline in CB patients.

In this study, CB severity was stratified using CAT sub-questionnaires and various cut-off values. Questionnaires for CB were developed by the American Thoracic Society in 1978 and they are frequently used to define the disease [16, 25, 35]. However, there may be recall bias during long-term evaluations and the definitions of CB are complicated; thus, other parameters have been used in some studies, including chronic cough, physician diagnosis, and the presence of cough and sputum for 3 months over > 1 year [12, 13, 36]. Symptom-based scores (e.g., SGRQ and CAT) have also been employed [11, 15, 17, 21, 27]. Such scoring systems allow symptom severity to be quantified based on cut-off values, rather than subjective definitions. In this study, CB was defined as both CAT1 and CAT2 scores ≥ 3, as initially recommended by Lim et al. [21]. This cut-off value results in similar proportions of CB among COPD patients, as determined using classically defined CB. In addition, Lim et al. showed that a CAT-based definition explained CT airway parameters, such as mean wall thickness and mean wall area. In a previous study, we showed that patients with CAT score-defined CB shared clinical characteristics and outcomes with patients who had classically defined CB [15]. While cut-off values have not been validated in other populations, a recent study based on Subpopulations and Intermediate Outcomes Measures in COPD Study (SPIROMICS) data suggested CAT1 and CAT2 cut-off scores of ≥ 2 [17]. Further studies are needed to validate a CAT score-based definition of CB in other populations.

Our study had two main limitations. First, the CAT definition of CB has been validated only in the Korean population [37, 38]; thus, as noted above, further studies are needed to support its general use. However, the CAT questionnaire is a universal tool for measuring quality of life in patients with COPD. Second, the KOCOSS cohort mostly consisted of patients examined and treated at tertiary hospitals; therefore, it may not represent the entire COPD population. The strengths of our study included its novel stratification of CB severity such that the patients’ clinical characteristics could be analyzed in relation to disease severity. In addition, lung function decline (based on 3-year follow-up data) was compared between non-CB patients and CB patients; it was also compared between patients with mild CB versus severe CB. Finally, our study included a large number of patients, drawn from a nationwide database that had been enrolling patients for 7 years at the time of data extraction.

## Conclusions

Our study compared clinical characteristics between non-CB patients and CB patients. Consistent with previous studies, we found that CB patients had poorer respiratory, exercise capacity, and psychological scores; reduced lung function; and more frequent exacerbations. Regression analysis showed that CB patients had more frequent moderate-to-severe exacerbations than did non-CB patients, based on a 1-year follow-up assessment. Using different CAT score cut-off scores, we distinguished mild CB from severe CB, then compared the clinical features of these two groups. Patients with severe CB had higher respiratory, exercise capacity, and psychological scores. At the 1-year follow-up assessment, patients with severe CB had more frequent severe exacerbations, as determined in a regression model. These results highlight the need for physicians to carefully monitor patients with severe symptoms.

## Supplementary Information

**Additional file 1.** **Table S1**. Difference of clinical characteristics between non-CB and CB in ever-smokers. **Table S2**. Difference of clinical characteristics between mild CB and severe CB in ever-smokers. **Table S3**. Frequency of exacerbations for CB compared with non-CB patients in ever-smokers. **Table S4**. Frequency of exacerbations for severe CB compared with mild CB in ever-smokers.

## Data Availability

The datasets supporting the conclusions of this article are available from the corresponding author on reasonable request.

## Abbreviations

COPD: Chronic obstructive pulmonary disease
CB: Chronic bronchitis
ACO: Asthma-COPD overlap
ATS: American Thoracic Society
CAT score: COPD Assessment Test score
KOCOSS: Korea COPD Subgroup Study
FEV_1_: Forced expiratory volume in 1 s
FVC: Forced vital capacity
CRF: Form of case report
BMI: Body mass index (BMI)
mMRC scale: Modified Medical Research Council Scale
6MWT: 6-Min walk distance test
BDI score: Beck Depression Inventory score
BAI score: Beck Anxiety Inventory score
FeNO: Fractional exhaled nitric oxide
IgE: Immunoglobulin E (IgE)
PFT: Pulmonary function test
DLco: Diffusion capacity of the lung for carbon monoxide
RV: Residual volume
TLC: Total lung capacity
CT: Computed tomography
LABA: Long-acting beta-agonist
LAMA: Long-acting muscarinic antagonist
ICS: Inhaled corticosteroid
SF-36 score: 36-Item Short Form Health Survey score
PROMIS-29: Patient-Reported Outcome Measurement Information System Scores
CARDIA study: Coronary Artery Risk Development in Young Development in Young Adults study

## References

[1] Global initiative for chronic obstructive lung disease (GOLD) guidelines, global strategy for the diagnosis, management and prevention of chronic obstructive lung disease. 2021; https://goldcopd.org/2021-gold-reports/.

[2] Agusti A (2013). Phenotypes and disease characterization in chronic obstructive pulmonary disease. Toward the extinction of phenotypes?. Ann Am Thorac Soc.

[3] Miravitlles M, Soler-Cataluna JJ, Calle M, Soriano JB (2013). Treatment of COPD by clinical phenotypes: putting old evidence into clinical practice. Eur Respir J.

[4] Corlateanu A, Mendez Y, Wang Y, Garnica RJA, Botnaru V, Siafakas N (2020). Chronic obstructive pulmonary disease and phenotypes: a state-of-the-art. Pulmonology.

[5] Fletcher C, Peto R (1977). The natural history of chronic airflow obstruction. BMJ.

[6] Dotan Y, So JY, Kim V (2019). Chronic bronchitis: where are we now?. Chronic Obstruct Pulm Dis.

[7] AbuDagga A, Sun SX, Tan H, Solem CT (2013). Healthcare utilization and costs among chronic bronchitis patients treated with maintenance medications from a US managed care population. J Med Econ.

[8] Pasquale MK, Sun SX, Song F, Hartnett HJ, Stemkowski SA (2012). Impact of exacerbations on health care cost and resource utilization in chronic obstructive pulmonary disease patients with chronic bronchitis from a predominantly Medicare population. Int J Chron Obstruct Pulmon Dis.

[9] Blanchette CM, Roberts MH, Petersen H, Dalal AA, Mapel DW (2011). Economic burden of chronic bronchitis in the United States: a retrospective case-control study. Int J Chron Obstruct Pulmon Dis.

[10] Kim V, Han MK, Vance GB, Make BJ, Newell JD, Hokanson JE, Hersh CP, Stinson D, Silverman EK, Criner GJ (2011). The chronic bronchitic phenotype of COPD: an analysis of the COPDGene Study. Chest.

[11] Kim V, Crapo J, Zhao H, Jones PW, Silverman EK, Comellas A, Make BJ, Criner GJ (2015). Comparison between an alternative and the classic definition of chronic bronchitis in COPDGene. Ann Am Thorac Soc.

[12] Vestbo J, Prescott E, Lange P (1996). Association of chronic mucus hypersecretion with FEV1 decline and chronic obstructive pulmonary disease morbidity. Copenhagen City Heart Study Group. Am J Respir Crit Care Med.

[13] Pallasaho P, Lundback B, Laspa SL, Jonsson E, Kotaniemi J, Sovijarvi AR, Laitinen LA (1999). Increasing prevalence of asthma but not of chronic bronchitis in Finland? Report from the FinEsS-Helsinki Study. Respir Med.

[14] Corhay JL, Vincken W, Schlesser M, Bossuyt P, Imschoot J (2013). Chronic bronchitis in COPD patients is associated with increased risk of exacerbations: a cross-sectional multicentre study. Int J Clin Pract.

[15] Choi JY, Yoon HK, Shin KC, Park SY, Lee CY, Ra SW, Jung KS, Yoo KH, Lee CH, Rhee CK (2019). CAT score and SGRQ definitions of chronic bronchitis as an alternative to the classical definition. Int J Chron Obstruct Pulmon Dis.

[16] Ferris BG (1978). Epidemiology Standardization Project (American Thoracic Society). Am Rev Respir Dis.

[17] Stott-Miller M, Müllerová H, Miller B, Tabberer M, El Baou C, Keeley T, Martinez FJ, Han M, Dransfield M, Hansel NN (2020). Defining chronic mucus hypersecretion using the CAT in the SPIROMICS cohort. Int J Chron Obstruct Pulmon Dis.

[18] Papaioannou M, Pitsiou G, Manika K, Kontou P, Zarogoulidis P, Sichletidis L, Kioumis IP (2014). COPD assessment test: a simple tool to evaluate disease severity and response to treatment. COPD.

[19] Lee SD, Huang MS, Kang J, Lin CH, Park MJ, Oh YM, Kwon N, Jones PW, Sajkov D (2014). The COPD assessment test (CAT) assists prediction of COPD exacerbations in high-risk patients. Respir Med.

[20] Mackay AJ, Donaldson GC, Patel AR, Jones PW, Hurst JR, Wedzicha JA (2012). Usefulness of the chronic obstructive pulmonary disease assessment test to evaluate severity of COPD exacerbations. Am J Respir Crit Care Med.

[21] Lim JU, Lee JH, Kim TH, Lee JS, Lee SD, Oh YM, Rhee CK (2018). Alternative definitions of chronic bronchitis and their correlation with CT parameters. Int J Chron Obstruct Pulmon Dis.

[22] Liang Y, Chen Y, Wu R, Lu M, Yao W, Kang J, Cai B, Zhou X, Liu Z, Chen P (2017). Chronic bronchitis is associated with severe exacerbation and prolonged recovery period in Chinese patients with COPD: a multicenter cross-sectional study. J Thorac Dis.

[23] Lahousse L, Seys LJM, Joos GF, Franco OH, Stricker BH, Brusselle GG (2017). Epidemiology and impact of chronic bronchitis in chronic obstructive pulmonary disease. Eur Respir J.

[24] Kim V, Criner GJ (2015). The chronic bronchitis phenotype in chronic obstructive pulmonary disease: features and implications. Curr Opin Pulm Med.

[25] Ferre A, Fuhrman C, Zureik M, Chouaid C, Vergnenegre A, Huchon G, Delmas MC, Roche N (2012). Chronic bronchitis in the general population: influence of age, gender and socio-economic conditions. Respir Med.

[26] Zhang W, Lu H, Peng L, Ren X, Lu Y, An L, Zhang H, Tan X, Sun X, Huang K (2015). Chronic bronchitis leads to accelerated hyperinflation in COPD patients during exercise. Respirology (Carlton, Vic).

[27] Kim V, Zhao H, Regan E, Han MK, Make BJ, Crapo JD, Jones PW, Curtis JL, Silverman EK, Criner GJ (2019). The St. George's Respiratory Questionnaire definition of chronic bronchitis may be a better predictor of COPD exacerbations compared with the classic definition. Chest.

[28] Mejza F, Gnatiuc L, Buist AS, Vollmer WM, Lamprecht B, Obaseki DO, Nastalek P, Nizankowska-Mogilnicka E, Burney PGJ (2017). Prevalence and burden of chronic bronchitis symptoms: results from the BOLD study. Eur Respir J.

[29] Guerra S, Sherrill DL, Venker C, Ceccato CM, Halonen M, Martinez FD (2009). Chronic bronchitis before age 50 years predicts incident airflow limitation and mortality risk. Thorax.

[30] Meek PM, Petersen H, Washko GR, Diaz AA, Klm V, Sood A, Tesfaigzi Y (2015). Chronic bronchitis is associated with worse symptoms and quality of life than chronic airflow obstruction. Chest.

[31] Choi JY, Yoon HK, Park SJ, Park YB, Shin KC, Na JO, Yoo KH, Jung KS, Kim YK, Rhee CK (2016). Chronic bronchitis is an independently associated factor for more symptom and high-risk groups. Int J Chron Obstruct Pulmon Dis.

[32] Choate R, Pasquale CB, Parada NA, Prieto-Centurion V, Mularski RA, Yawn BP (2020). The burden of cough and phlegm in people with COPD: a COPD patient-powered research network study. Chronic Obstruct Pulm Dis.

[33] Kalhan R, Dransfield MT, Colangelo LA, Cuttica MJ, Jacobs DR, Thyagarajan B, Estepar RSJ, Harmouche R, Onieva JO, Ash SY (2018). Respiratory symptoms in young adults and future lung disease: the CARDIA lung study. Am J Respir Crit Care Med.

[34] Prescott E, Lange P, Vestbo J (1995). Chronic mucus hypersecretion in COPD and death from pulmonary infection. Eur Respir J.

[35] de Oca MM, Halbert RJ, Lopez MV, Perez-Padilla R, Talamo C, Moreno D, Muino A, Jardim JR, Valdivia G, Pertuze J (2012). The chronic bronchitis phenotype in subjects with and without COPD: the PLATINO study. Eur Respir J.

[36] Hurst JR, Vestbo J, Anzueto A, Locantore N, Mullerova H, Tal-Singer R, Miller B, Lomas DA, Agusti A, Macnee W (2010). Susceptibility to exacerbation in chronic obstructive pulmonary disease. N Engl J Med.

[37] Kwon N, Amin M, Hui DS, Jung KS, Lim SY, Ta HD, Thai TTL, Yunus F, Jones PW (2013). Validity of the COPD assessment test translated into local languages for Asian patients. Chest.

[38] Hwang YI, Jung KS, Lim SY, Lee YS, Kwon NH (2013). A validation study for the Korean Version of Chronic Obstructive Pulmonary Disease Assessment Test (CAT). Tuberc Respir Dis.

