# Supplementary material for: Comparison of clinical characteristics between chronic bronchitis and non-chronic bronchitis in patients with chronic obstructive pulmonary disease
Source: BMC Pulm Med. 2022 Feb 20;22:69. doi: 10.1186/s12890-022-01854-x (PMC8858532; doi:10.1186/s12890-022-01854-x)
Supplement: Supplementary file 1 — Additional file 1. Table S1. Difference of clinical characteristics between non-CB and CB in ever-smokers. Table S2. Difference of clinical characteristics between mild CB and severe CB in ever-smokers. Table S3. Frequency of exacerbations for CB compared with non-CB patients in ever-smokers. Table S4. Frequency of exacerbations for severe CB compared with mild CB in ever-smokers. [file 12890_2022_1854_MOESM1_ESM.docx]

**SUPPLEMENTARY INFORMATION**

**Comparison of clinical characteristics between chronic bronchitis and non- chronic bronchitis in patients with chronic obstructive pulmonary disease**

Authors’ full names:

Joon Young Choi, MD, PhD^1^; Hyoung Kyu Yoon, MD, PhD^2^; Sang Yeub Lee, MD, PhD^3^; Jin Woo Kim, MD, PhD^4^; Hye Sook Choi, MD, PhD^5^; Yu-Il Kim, MD, PhD^6^; Ki-Suck Jung, MD, PhD^7^; Kwang Ha Yoo, MD, PhD^8^; Woo Jin Kim, MD, PhD^9^; Chin Kook Rhee, MD, PhD^10^

Authors’ affiliation(s):

^1^Division of Pulmonary and Critical Care Medicine, Department of Internal Medicine, Incheon St. Mary’s Hospital, College of Medicine, The Catholic University of Korea, Seoul, Republic of Korea

^2^Division of Pulmonology, Critical Care and Sleep Medicine, Department of Internal Medicine, Yeouido St. Mary's Hospital, College of Medicine, The Catholic University of Korea, Seoul, Republic of Korea

^3^Division of Pulmonology, Allergy and Critical Care Medicine, Department of Internal Medicine, Korea University Anam Hospital, Korea University College of Medicine, Seoul, Republic of Korea.

^4^Division of Pulmonology and Critical Care Medicine, Department of Internal Medicine, Uijeongbu St. Mary's Hospital, College of Medicine, The Catholic University of Korea, Uijeongbu, Republic of Korea

^5^Division of Pulmonary, Allergy and Critical Care Medicine, Department of Internal Medicine, Kyung Hee University Hospital, Seoul, Republic of Korea.

^6^Division of Pulmonary Medicine, Department of Internal Medicine, Chonnam National University Hospital, Gwangju, Republic of Korea

^7^Division of Pulmonary, Allergy and Critical Care Medicine, Hallym University Sacred Heart Hospital, Hallym University Medical School, Anyang, Republic of Korea.

^8^Division of Pulmonary, Allergy and Critical Care Medicine, Department of Internal Medicine, Konkuk University School of Medicine, Seoul, Republic of Korea

^9^Department of Internal Medicine and Environmental Health Center, School of Medicine, Kangwon National University, Chuncheon, Republic of Korea

^10^Division of Pulmonary and Critical Care Medicine, Department of Internal Medicine, Seoul St. Mary’s Hospital, College of Medicine, The Catholic University of Korea, Seoul, Republic of Korea

**The names of ethics committees**

Gacheon University Gil Medical Center, Hallym University Kangnam Sacred Heart Hospital, Gangnam Severance Hospital, Kyung Hee University Hospital at Gangdong, Hallym University Kangdong Sacred Heart Hospital, Kangbuk Samsung Hospital, Kangwon National University Hospital, Konkuk University Hospital, Konkuk University Chungju Hospital, Kyungpook National University Hospital, Gyeongsang National University Hospital, Korea University Guro Hospital, Korea University Anam Hospital, Seoul Eulji Hospital, Dongguk University Gyeongju Hospital, Dongguk University Ilsan Hospital, Keimyung University Dongsan Medical Center, Dong-A University Hospital, Hallym University Dongtan Sacred Heart Hospital, Pusan National University Hospital, Inje University Busan Paik Hospital, The Catholic University of Korea Bucheon St Mary’s Hospital, Soonchunhyang University Hospital Bucheon, Seoul National University Bundang Hospital, Bundang CHA Hospital, Seoul Metropolitan Government Seoul National University Bora-mae Medical Center, Samsung Medical Center, Soonchunhyang University Hospital Seoul, The Catholic University of Korea Seoul St Mary’s Hospital, The Catholic University of Korea St Paul’s Hospital, The Catholic University of Korea St Vincent’s Hospital, Severance Hospital, Asan Medical Center, Ajou University Hospital, The Catholic University of Korea Yeouido St Mary’s Hospital, The Catholic University of Korea Uijeongbu St Mary’s Hospital, Yeungnam University Medical Center, Ulsan University Hospital, Wonkwang University Sanbon Hospital, Wonju Severance Christian Hospital, Ewha Womans University Mokding Hospital, Incheon St Mary’s Hospital, Inha University Hospital, Chonnam National University Hospital, Chonbuk National University Hospital, Jeju National University Hospital, Soonchunhyang University Hospital Cheonan, Hallym University Chuncheon Sacred Heart Hospital, Hallym University Sacred Heart Hospital, and Hanyang University Guri Hospital.

We also received approval from each center to use their subjects’ clinical records for the study while maintaining the confidentiality of the data

Table S1. Difference of clinical characteristics between non-CB and CB in ever-smokers

|  | Non-CB  (n=1413, 77.0%) | CB  (n=422, 23.0%) | P-value |
| --- | --- | --- | --- |
| Age | 69.3 ± 7.6 | 68.2 ± 7.7 | 0.01 |
| Sex (male) | 1372 (97.1%) | 410 (97.2%) | 1.00 |
| Smoking Hx |  |  | <0.01 |
| -Ex-smoker | 1043 (73.8%) | 262 (62.1%) |  |
| -Current smoker | 370 (26.2%) | 160 (37.9%) |  |
| BMI | 23.0 ± 3.4 | 22.5 ± 3.4 | <0.01 |
| mMRC | 1.2 ± 0.8 | 1.6 ± 1.0 | <0.01 |
| CAT score | 12.0 ± 6.4 | 22.6 ± 7.3 | <0.01 |
| 6MWT | 384.1 ± 119.4 | 370.5 ± 113.0 | 0.07 |
| BDI score | 6.0 ± 7.3 | 9.8 ± 9.7 | <0.01 |
| BAI score | 3.8 ± 5.3 | 7.1 ± 9.3 | <0.01 |
| Asthma Hx | 387 (27.6%) | 133 (31.7%) | 0.11 |
| ACO | 159 (20.7%) | 53 (23.6%) | 0.40 |
| Emphysema | 334 (46.6%) | 123 (52.8%) | 0.12 |
| Bronchiectasis | 73 (10.2%) | 30 (12.9%) | 0.31 |
| GOLD stage |  |  | <0.01 |
| - I | 155 (11.0%) | 23 (5.5%) |  |
| - II | 749 (53.0%) | 195 (46.2%) |  |
| - III | 410 (29.0%) | 155 (36.7%) |  |
| - IV | 98 (6.9%) | 49 (11.6%) |  |
| postBD FEV1 (L) | 1.7 ± 0.6 | 1.6 ± 0.6 | <0.01 |
| postBD FVC (L) | 3.4 ± 0.8 | 3.3 ± 0.8 | 0.04 |
| FEV1/FVC | 50.6 ± 12.8 | 47.1 ± 12.7 | <0.01 |
| DLco | 64.0 ± 20.6 | 60.5 ± 20.5 | <0.01 |
| RV/TLC | 0.4 ± 0.1 | 0.4 ± 0.1 | <0.01 |
| FeNO | 27.6 ± 17.1 | 27.8 ± 19.3 | 0.95 |
| Blood eosinophil count | 232.9 ± 267.6 | 230.8 ± 223.2 | 0.88 |
| IgE | 255.0 ± 386.1 | 227.5 ± 313.2 | 0.33 |
| Medications |  |  |  |
| - LABA or LAMA | 368 (26.0%) | 90 (21.3%) | 0.06 |
| - LABA/LAMA | 268 (19.0%) | 60 (14.2%) | 0.03 |
| - ICS/LABA | 166 (11.7%) | 49 (11.6%) | 1.00 |
| - ICS/LABA/LAMA | 304 (21.5%) | 108 (25.6%) | 0.09 |
| M-S exacerbation (Y/N) | 390 (37.9%) | 152 (52.2%) | <0.01 |
| MS exacerbation  (Frequency) | 0.9 ± 1.9 | 1.7 ± 2.6 | <0.01 |
| S exacerbation (Y/N) | 104 (10.1%) | 40 (13.7%) | 0.10 |
| S exacerbation (Frequency) | 0.1 ± 0.5 | 0.3 ± 1.0 | <0.01 |

Table S2. Difference of clinical characteristics between mild CB and severe CB in ever-smokers

|  | Mild CB  (n=287, 68.0%) | Severe CB  (n=135, 32.0%) | P-value |
| --- | --- | --- | --- |
| Age | 68.4 ± 7.8 | 67.7 ± 7.5 | 0.37 |
| Sex (male) | 297 (97.2%) | 131 (97.0%) | 1.00 |
| Smoking Hx |  |  | 0.12 |
| -Ex-smoker | 186 (64.8%) | 76 (56.3%) |  |
| -Current smoker | 101 (35.2%) | 59 (43.7%) |  |
| BMI | 22.6 ± 3.3 | 22.2 ± 3.6 | 0.31 |
| mMRC | 1.5 ± 0.9 | 1.9 ± 1.0 | <0.01 |
| CAT score | 20.6 ± 6.5 | 27.1 ± 6.8 | <0.01 |
| 6MWT | 379.2 ± 112.5 | 351.6 ± 112.3 | 0.04 |
| BDI score | 8.7 ± 9.0 | 11.7 ± 10.7 | 0.03 |
| BAI score | 5.4 ± 7.4 | 10.8 ± 11.7 | <0.01 |
| Asthma Hx | 88 (30.9%) | 45 (33.6%) | 0.66 |
| ACO | 39 (26.5) | 14 (17.9%) | 0.20 |
| Emphysema | 84 (52.8%) | 39 (52.7%) | 1.00 |
| Bronchiectasis | 18 (11.3%) | 12 (16.2%) | 0.41 |
| GOLD stage |  |  | 0.60 |
| - I | 15 (5.2%) | 8 (5.9%) |  |
| - II | 138 (48.1%) | 57 (42.2%) |  |
| - III | 104 (36.2%) | 51 (37.8%) |  |
| - IV | 30 (10.5%) | 19 (14.1%) |  |
| postBD FEV1 (L) | 1.6 ± 0.6 | 1.5 ± 0.5 | 0.21 |
| postBD FVC (L) | 3.3 ± 0.8 | 3.2 ± 0.8 | 0.24 |
| FEV1/FVC | 47.1 ± 12.9 | 47.0 ± 12.2 | 0.89 |
| DLco | 61.2 ± 20.9 | 58.9 ± 19.4 | 0.33 |
| RV/TLC | 0.4 ± 0.1 | 0.5 ± 0.1 | 0.36 |
| FeNO | 30.9 ± 21.1 | 18.6 ± 6.6 | 0.01 |
| Blood eosinophil count | 226.0 ± 214.8 | 240.6 ± 240.4 | 0.57 |
| IgE | 223.3 ± 292.1 | 216.0 ± 353.8 | 0.73 |
| Medications |  |  |  |
| - LABA or LAMA | 65 (22.6%) | 25 (18.5%) | 0.40 |
| - LABA/LAMA | 41 (14.3%) | 19 (14.1%) | 1.00 |
| - ICS/LABA | 32 (11.1%) | 17 (12.6%) | 0.79 |
| - ICS/LABA/LAMA | 71 (24.7%) | 37 (27.4%) | 0.64 |
| M-S exacerbation (Y/N) | 100 (49.3%) | 52 (59.1%) | 0.16 |
| MS exacerbation  (Frequency) | 1.5 ± 2.4 | 2.1 ± 3.1 | 0.16 |
| S exacerbation (Y/N) | 19 (9.4%) | 21 (23.9%) | <0.01 |
| S exacerbation (Frequency) | 0.2 ± 0.9 | 0.5 ± 1.2 | 0.05 |

Table S3. Frequency of exacerbations for CB compared with non-CB patients in ever-smokers

|  | Moderate-to-severe exacerbation | | | Severe exacerbation | | |
| --- | --- | --- | --- | --- | --- | --- |
|  | OR | 95%CI | p-value | OR | 95%CI | p-value |
| CB | 1.52 | 1.23-1.89 | <0.01 | 1.52 | 1.02-2.28 | 0.04 |
| Age | 1.00 | 0.99-1.01 | 0.90 | 0.99 | 0.97-1.02 | 0.70 |
| Sex (age) | 0.81 | 0.47-1.44 | 0.45 | 0.44 | 0.12-1.38 | 0.19 |
| Smoking Hx | 0.94 | 0.76-1.17 | 0.60 | 0.76 | 0.49-1.18 | 0.23 |
| FEV1 | 0.36 | 0.30-0.43 | <0.01 | 0.17 | 0.11-0.25 | <0.01 |

Table S4. Frequency of exacerbations for severe CB compared with mild CB in ever-smoker

|  | Moderate-to-severe exacerbation | | | Severe exacerbation | | |
| --- | --- | --- | --- | --- | --- | --- |
|  | OR | 95%CI | p-value | OR | 95%CI | p-value |
| Severe CB | 1.24 | 0.86-1.80 | 0.26 | 2.56 | 1.22-5.59 | 0.01 |
| Age | 1.00 | 0.98-1.03 | 0.75 | 1.01 | 0.96-1.06 | 0.69 |
| Sex (male) | 1.52 | 0.46-6.49 | 0.51 | 0.36 | 0.01-12.3 | 0.58 |
| Smoking Hx | 1.90 | 0.63-1.31 | 0.60 | 0.70 | 0.31-1.59 | 0.40 |
| FEV1 | 0.36 | 0.26-0.50 | <0.01 | 0.09 | 0.03-0.19 | <0.01 |
